# Is neuroimaging measuring information in the brain?

**DOI:** 10.3758/s13423-016-1002-0

**Published:** 2016-02-01

**Authors:** Lee de-Wit, David Alexander, Vebjørn Ekroll, Johan Wagemans

**Affiliations:** 1 Institute of Continuing Education, University of Cambridge, Cambridge, UK; 2 Psychology and Language Sciences, University College London, London, UK; 3 Laboratory of Experimental Psychology, University of Leuven, Leuven, Belgium

**Keywords:** Cognitive neuroscience, Neuroimaging

## Abstract

Psychology moved beyond the stimulus response mapping of behaviorism by adopting an information processing framework. This shift from behavioral to cognitive science was partly inspired by work demonstrating that the concept of information could be defined and quantified (Shannon, 1948). This transition developed further from cognitive science into cognitive neuroscience, in an attempt to measure information in the brain. In the cognitive neurosciences, however, the term information is often used without a clear definition. This paper will argue that, if the formulation proposed by Shannon is applied to modern neuroimaging, then numerous results would be interpreted differently. More specifically, we argue that much modern cognitive neuroscience implicitly focuses on the question of how we can interpret the activations we record in the brain (experimenter-as-receiver), rather than on the core question of how the rest of the brain can interpret those activations (cortex-as-receiver). A clearer focus on whether activations recorded via neuroimaging can actually act as information in the brain would not only change how findings are interpreted but should also change the direction of empirical research in cognitive neuroscience.

## Everybody knows what information is

The word ‘information’ is often used in a way that implicitly assumes an obvious and pre-defined meaning. Just like the concept of attention, however, ‘information’ means very different things to different people. The intuition that ‘everybody knows what information is’ is therefore problematic. Indeed, without a clear definition, the search for ‘information’ or ‘representations’ in the brain could potentially be as misleading as the search for the ‘vital forces’ in biology or ‘impetus’ in classical mechanics.

The intention of this paper is not to undermine the potential of using neuroimaging to measure information, but rather to make more explicit the assumptions that may lead us astray in that endeavor. We will argue that most neuroimaging (implicitly) focuses on interpreting physical signals in the brain from the perspective of an external experimenter, whereas the key question for neuroscience should be how (or whether) those signals are used by the rest of the brain. In light of Shannon’s (1948) information theory, we will argue that information in neuroscience is often measured with an implicit ‘experimenter-as-receiver’ assumption, rather than thinking in terms of ‘cortex-as-receiver’. For example, many studies report differential responses in the brain (from single cells, EEG, fMRI, etc.) as the discovery of ‘the neural representation of X’ or ‘revealing the neural code underlying Y’ without ever providing evidence that those recorded responses reflect differences in activity that can actually be used (received or decoded) by other areas of the brain. This is a problem because information is not a static property inherent to a physical response: It is only when physical responses can be shown to be used by the brain that we have positive evidence that a physical signal acts as information. A neuron might fire vigorously every time an organism is presented with a visual object (for example), and as an external observer it is easy for us to assume that the vigorous firing of that neuron informs the rest of the system about that object. If, however, the rate at which that neuron fires never influences any other processes in the brain, then that firing rate cannot be part of the neural code underlying the representation of that object. It could be that the firing rate is an artifact or by-product, and that the actual information is contained in the phase at which that neuron fires. Most importantly, the only way we can test this is by following the dynamics of what causes what in the brain, but this is quite different from the focus of most neuroscience and neuroimaging. We believe our understanding of what information is lies at the heart of a shift in emphasis that is needed in cognitive neuroscience.

## The nature of measurement

What does it mean to measure information in the brain? When we record neural activity after the presentation of a stimulus, can we call that activity the neural representation of that stimulus? What technique provides the best measure of information? Are single-cell recordings a more direct measure of information than fMRI or EEG? Does fMRI sometimes provide a better measure of information by looking at activity at a larger scale? Can the more precise timing of EEG and MEG sometimes provide a better measure of information in the brain?

To answer these questions, we need to step back, not just to consider what information is but also to consider any process of measurement. Critically, all measurement is indirect. We always have to measure via some instrument, which necessarily entails assumptions about how that device works (Kuhn, 1961). Consider for example how we ‘measure’ the existence of the nucleus of an atom. In his classical experiments, Rutherford launched helium molecules at thin sheets of gold and observed that a small proportion of these helium molecules would not follow their original path, but seemed to be deflected onto a new path. These deflections were assumed to happen because the helium nucleus would occasionally collide with the very small nucleus of the gold atoms. In this way, the structure of the atom was not directly observed but inferred from its effects on the measurement apparatus. All measurement is of this type (Kuhn, 1961). We make inferences about the structure or nature of things based on indirect observations. The inference that an atom contains a relatively tiny nucleus is now so plausible that we regard it as a fact, and Rutherford’s experiments may, in retrospect, seem like a direct means of measuring an apparently known feature of our physical world.

If all measurement is actually an inference, what inference enables us to posit the existence of information? Shannon provided an explicit formal description of this inference by quantifying the amount of information that can be conveyed over a noisy channel. This formulation was made up of a number of steps, including a transmitter, a channel (with signal and noise) and a receiver. The focus of Shannon’s formulation was on the signal and noise of the channel, but he made it clear that whatever was sent over the channel would need to be decoded by a receiver. Thus, in Shannon’s formulation, the quantification of information over a channel was contingent on the existence of a ‘receiver’. The importance of a receiver in Shannon’s formulation seems to be neglected in modern neuroscience, perhaps because, for the communication systems which he was considering, he merely stated that (p. 2) ‘The receiver ordinarily performs the inverse operation of that done by the transmitter’, and he did not elaborate much further on this point. We will give two examples, a simple thermostat and a process of encryption, to illustrate that it is impossible to meaningfully consider something to be information without considering how (or whether) a signal can be decoded by a receiver.

Whilst philosophers debate whether a thermostat can be considered conscious, it most certainly provides a concrete example for thinking about Shannon’s conception of an information processing system. In Shannon’s terms, the temperature gauge could take the role of the transmitter, the channel could be a current-carrying wire, and the heating device could be the receiver which turns on or off contingent upon the state of the electrical current in the wire. The electrical current in the wire can be regarded as information in this system because it is decoded and interpreted by a receiver. Critically, however, we cannot make the inference that an electrical current is information unless it is causally influenced by a transmitter, and exerts a causal influence on a receiver. We will argue that a lot of neuroscience focuses on quantifying the potential sources of information without giving enough consideration to how (or whether) that potential information might actually be decoded by a receiver. To illustrate the necessity of this, we can envisage a different set-up for our cartoon information processing system in our thermostat, where rather than remaining on when there is a current, and turning off when there is none, the state of the heater (on or off) changes every time a current is passed through the wire. In this simple example, the information content is not just determined by physical flow of electrons in the wire but also by the current state of the receiver. Indeed, we could envisage another system where the existence of a constant current, by itself, cannot be decoded by the receiver in any meaningful way, but rather the receiver turns the radiator on and off depending on the precise timing of pulses in the current. Information, in our simple thermostat example, is therefore not inherent to the physical activity (current in the wire) but one has to think about how any measurable aspects of the current will be used by the receiver. To combine a quote attributed to Bateson, with Shannon’s ideas, for information to truly be information, it has to be a *difference that makes a difference to a receiver*. Critically, therefore, when reverse-engineering any device, one cannot simply measure a physical property (the flow of electrons in a wire, the firing of a neuron, a pattern of activity measured with fMRI) and assume that one has measured information. Rather, you have to construct a model of how that physical activity can be used as a signal by a receiver.

In other words, there is no such thing as *objective* information, because it is always *subject* to interpretation by a receiver. Another example which makes the subjective nature of information explicit is encryption. Good encryption algorithms will make the target information appear as noise to an observer or receiver who does not have the correct decryption key. When the observer has the correct decryption key, the information in the message is interpretable. So, whether the message is noise or information depends on the properties of the receiver. In this case, the critical question is whether the receiver is in possession of the correct decryption key. Without the key, there is no immediate way to tell whether a message is signal or noise. For example, we can take a message, and encrypt it by combining it with some noise that has been generated for the purpose of creating a one-time encryption key. The message can be encrypted by the transmitter by multiplying each bit in the message by its corresponding paired bit in the key made of random noise. The sender gives the noise key to the receiver, and so the noise is now knowledge owned by the receiver. At a later date, the sender sends the encrypted message across a public channel. It appears as noise to all receivers except the one with the correct key. Now the noise we generated is the key that enables the observer to decrypt the message. What counts as signal and what counts as noise very much depends on the correct model of the interaction between the sender, the channel and the receiver. We will argue that the purpose of neuroscience is to find the ‘correct model of interaction’ for the case of the brain. Any measurement of information in the brain’s system very much depends on the model used for its correct interactions, and inferences about what is signal (and equally critically what is noise) cannot be made without considering this. Without doing so, there is a risk that one could assume that recorded neural activity contains objective information that can be read out in a direct empirical manner.

## Single-cell recordings do not directly measure information

Perhaps the best way to get to the heart of the issue is to consider Hubel and Wiesel’s recording from the primary visual cortex, in which they found cells that would fire when the animal was presented with stimuli that we would call edges (Hubel & Wiesel, 1959). This result is often interpreted as evidence that the primary visual cortex represents edges, or, more boldly, that individual neurons in the primary visual cortex represent edges. Whether or not a single cell can code for anything, however, depends entirely on the underlying coding model. To say that the firing of one cell represents an edge assumes that other parts of the brain can explicitly make use of the firing of this one cell in causing further processing, and ultimately in generating behavior. It is entirely plausible, however, that some other feature of the physical activity is used at further stages of processing. For example, the actual information could be carried in:

1. the pattern of activity across a large number of cells or a population code (Pouget, Dayan, & Zemel, 2000);
2. the timing of the first wave of spikes (Thorpe, Delorme, & Van Rullen, 2001);
3. the timing or phase of continuous activity (Schyns, Thut, & Gross, 2011);
4. synchrony across a population (Singer, 1999);
5. or a combination of these.

That is not to say that it is impossible (or even implausible) that single cells are ever used as symbolic representations (Bowers, 2009). Indeed, part of the excitement around single-cell recording techniques stemmed from the fact that the firing of individual cells could sometimes provide very good predictions of how the animal would respond on a given task. However, there are many reasons why the activity of a given cell might correlate with the behavioral output of the organism that entirely depend upon the coding model that has been assumed. To its credit, some of the most influential and foundational work in linking neural activity to perceptual decisions was very clear about this, and explicitly referred to ‘neural *correlates* of a perceptual decision’ (emphasis added; Newsome, Britten, & Movshon, 1989), rather than the ‘neural *representation* of a perceptual decision’.

The problem in assuming that a given neural response is an explicit representation of something is also highlighted by the breadth of other things that V1 cells could be representing. Koenderink (2012), for example, questions what exactly is an edge, in the absence of a circular definition based upon what V1 cells respond to. Koenderink argues that the intuitive appeal of a functional role for V1 cells as edge detectors could mislead us from their actual function, for example, as a filter whose responses sometimes approximate what we might (mistakenly) assume to be an edge detector. Edge-detectors may also be better understood as the most efficient means of encoding the pattern of two-dimensional input typically encountered in natural images (Olshausen & Field, 1996), a relabeling that leads to a different set of questions. Others have argued that responses in V1 are sometimes not driven by bottom–up input alone. More specifically, Rao and Ballard (1999) have argued that, rather than reflecting a representation of a certain kind of input, ‘end-stopped’ neurons in fact signal (provide information regarding) the violation of a prediction of the existence of a longer line based on feedback from higher areas. Others have argued that receptive field responses in V1 are organized in terms of contextual maps of textures that appear in the visual field (Alexander & Van Leeuwen, 2010). This again provides a very different interpretation, such that the classical edge-detection response within the receptive field in a standard V1 experiment actually arises due to an unusual absence of contextual cues. Indeed, rather than isolating the representational building blocks of visual perception, it may be that neurons in V1 simply respond in qualitatively different ways when presented with more complex spatio-temporal signals that are closer to the visual input patterns (or natural scene statistics) that normally impinge upon our sensory apparatus (Olshausen & Field, 2005).

Recordings made from V1 can therefore be interpreted in different ways. Although this should not be surprising, it clarifies that (just like measures of the current in the wire of our thermostat above) single-cell recordings do not provide a direct measure of information. In terms of Shannon’s framework, we cannot infer whether the firing of a neuron in V1 represents anything without considering the receiver of that information.

In the literature, the extent to which responses of V1 are used by the rest of the brain is often evaluated by comparing it to the behavioral performance of the participant. This certainly helps to increase the confidence to infer that a response is used by the rest of the system, but the motor cortex responsible for generating the end signal to press one button or another is very unlikely to literally be the receiver of the response the experimenter records in V1. Looking at the anatomy, it seems that, whilst many areas project back to V1, V1 itself only sends input to a smaller number of visual areas (Kravitz, Saleem, Baker, Ungerleider, & Mishkin, 2013). From this perspective, V2 or MT are plausible ‘receivers’ for V1, but the motor cortex probably is not. The key question then becomes: Does the firing of a neuron in V1 ever cause activity in V2 (or any other receiver for V1)? If it does not, it is a difference that never makes a difference. It is not information, even if it correlates with behavior. It could be that the firing of a given V1 neuron only evokes a response in V2 (for example) if a particular pattern of neurons in V1 are active. In this case, the information is contained not in the response of any one cell but in the pattern of responses across cells. Similarly, one could find that the firing of neurons in V1 causes no further activity in V2, unless it has a very precise timing pattern. There are therefore multiple ‘channels’ over which V1 could actually be communicating, using a variety of possible codes, and we need to focus on the ‘cortex-as-receiver’ to track the causal dynamics from one area to the next to establish whether a measured response is indeed information used by the rest of the brain.

## Can fMRI decode information?

In the previous section, we saw that we cannot take for granted that information is conveyed via a particular channel (firing rate, spike timing, population codes, etc.), and that in order to identify the channel used by the brain, we need to consider how physical signals might be decoded by a receiver. Recent advances in fMRI analysis have reinforced this point by highlighting that much more potential information exists in the pattern of neural activity across voxels than had previously been assumed (Haxby et al., 2001; Haynes & Rees, 2006; Haynes & Rees, 2005; Kamitani, Tong, & Tong, 2005). Due to the noisy and variable nature of the signals recorded with fMRI, researchers had long assumed that one could find the clearest signals by taking the average of the activity levels over a large number of voxels. More recent Multi-Voxel-Pattern-Analysis (MVPA) techniques make clear, however, that spatial averaging can sometimes obscure potential signals contained in the pattern of activation across voxels. One such technique that has proved very influential involves the use of Support Vector Machines to train linear classifiers to combine potential signals across voxels. The increasing use of MVPA techniques illustrates the importance of considering how a receiver will decode information, and makes explicit that the amount of information potentially available is fundamentally contingent on the decoding method available.

It is typically assumed that any information that can be decoded from fMRI will also be available to the rest of the brain. Under this assumption, MVPA studies are often employed to identify the ‘neural representation’ of different cognitive and perceptual states. However, just as the firing of a single neuron cannot, without adequate justification, be assumed to represent anything, so too we need to acknowledge that the pattern of activation measured with fMRI might not represent anything to the rest of the brain. At the same time, we also need to remember that a failure to identify potential information with MVPA in a given area of the brain does not necessarily mean that there is no information available to the rest of the brain based on the activity in that area, as the information could be represented via a channel that is not detectable using fMRI MVPA.

In the literature, however, a failure to successfully decode a certain perceptual or cognitive distinction is sometimes interpreted as direct evidence that an area of the brain does not code for, or represent, that distinction. To pick an example (although there are many others), Kravitz, Peng and Baker (2011) used data from fMRI-MVPA to make the case that the Parahippocampal Place Area (PPA) represents the spatial layout of visual scenes but not their semantic content. Kravitz et al. demonstrated that the different spatial layouts of different visual scenes could be decoded from patterns of activation measured using fMRI, but the semantic content of those scenes could not. Their result does potentially provide some evidence to support the inference that PPA represents spatial layout and not semantics, but it could also be the case that semantics might be represented (and evoke differential neural responses) using a channel that is not observable using fMRI-MVPA. For example, it could be that semantic information is represented by a very sparse code in which only a very few neurons responded to different semantic categories (Bowers, 2009). Furthermore, even if such semantic information was represented by the firing rate of neurons using a large-scale population code, it might still not be decoded using MVPA. To understand why this is the case, we need to step back and consider why successful decoding is possible at all.

The first demonstration that a linear classifier could reveal more potential information than was evident in the average activation level across voxels was based on the classification of visual orientation in the primary visual cortex. As reviewed in the previous section, we have long known that cells in V1 in the primate visual system respond differently to different orientations. Given that these different neural responses to different orientations are distributed across a very large area of cortex, it was previously assumed that the coarse spatial scale of fMRI was insensitive to these differences. Indeed, a typical (3 × 3 mm) voxel in V1 contains something in the order of 40,000 neurons (Olman, 2015), so one might assume that each voxel would contain cells with a mixture of different orientation responses, and thus respond (at the level of the whole voxel) equally to all orientations. However, Kamitani and Tong (2005) observed that some voxels had a slight bias for one orientation or another. The differential responses of individual voxels was not necessarily informative alone, but when combining the weak signals from voxels across V1, Kamitani and Tong were able to successfully decode which orientation was being presented to the participant. Kamitani and Tong originally assumed that the weak bias in individual voxels was caused by the clustering of cells with different firing rates to different orientations into so-called ‘orientation columns’ (i.e., anatomical clusters of cells with similar response properties to different orientations). The existence of ‘orientation columns’ means that responses to different orientations are unevenly distributed over V1, and that similar responses cluster together. This uneven distribution could be the reason why within 3 × 3 mm voxels there might be a slight bias for one orientation over another. Since Kamitani and Tong’s original observation, there has been some debate as to whether the bias in responses to different orientations is caused by the clustering of different responses into orientation columns (Alink, Krugliak, Walther, & Kriegeskorte, 2013; Mannion, McDonald, & Clifford, 2009; Swisher et al., 2010), or due to larger-scale anatomical biases in the distribution of responses to different orientations across V1 (Freeman, Brouwer, Heeger, & Merriam, 2011; Freeman, Heeger, & Merriam, 2013; Op de Beeck, 2010). We shall return to this debate shortly, but for now it is sufficient to note that an uneven anatomical distribution of response properties is required for biases to exist at the level of individual voxels.

The necessity of this uneven anatomical distribution of different responses in supporting decoding is concretely illustrated in a recent study by Dubois, Berker and Tsao (2015), who demonstrated that fMRI-MVPA cannot decode the identity of a face in a particular area of the brain, but that neurons in that area show differential firing rates to different faces. Dubois et al. argued that the inability to successfully decode facial identity using fMRI MVPA is likely caused by a relatively homogeneous anatomical distribution of neurons that respond to different orientations. It would be tempting to conclude from this study that this area of the brain does contain information about facial identity (evident in the firing of individual neurons) but that this information is not evident using fMRI. Again, however, we would need to demonstrate that this aspect of neural activity (firing of individual neurons in response to individual faces) could be received and used by other areas of the brain to conclude that it was actual information.

The same point should also be clear when fMRI-MVPA is successful in revealing ‘potential information’. Revealing potential sources of information is clearly valuable, but we need to critically investigate how that activity could be used by other areas of the brain to conclude that it is ‘actual information’ used by the brain. Up to this point, we have focused on the reasons why decoding might not be successful (when information might be present). We will now explain in more detail why successful decoding (with experimenter-as-receiver) cannot be assumed to measure actual information (with cortex-as-receiver). In the case of decoding of orientation in V1, we briefly touched on the debate between competing theories regarding why responses to different orientations are distributed in a way that leads to a bias towards one orientation or another within individual voxels (that subsequently enables decoding). Regardless of whether this bias is due to clustering into orientation columns (Alink, Krugliak, Walther, & Kriegeskorte, 2013; Mannion, McDonald, & Clifford, 2009; Swisher et al., 2010) or larger-scale biases across V1 (Freeman, Brouwer, Heeger, & Merriam, 2011; Freeman, Heeger, & Merriam, 2013; Op de Beeck, 2010), it should not be taken for granted that the pattern of activity that enables successful decoding (with experimenter-as-receiver) is something that can be used by the rest of the brain (cortex-as-receiver). If we consider the further processing of orientation signals, for example, the next stage of information processing (V2) only samples information (receives inputs) from a relatively small region of V1 (Dumoulin & Wandell, 2008; Levitt, Kiper, & Movshon, 1994; Smith, Singh, Williams, & Greenlee, 2001) corresponding to its receptive field. In contrast, successful decoding with fMRI often samples across (uses inputs from) the whole of V1. Thus, it is questionable whether any ‘receiver’ in V2 could combine the inputs used to identify potential information in V1 using fMRI-MVPA. Of course, this is not to say that there are no areas of the brain that might be able to sample across V1 (especially as receptive fields become larger at further stages of the visual system), but we should not take for granted that something that we can decode with the ‘experimenter-as-receiver’ is accessible with the ‘cortex-as-receiver’. Successful decoding certainly shows that potential information is available, but it does not prove that any patterns used for decoding are used as information by the brain. A good illustration of this point is provided by the existence of retinotopic maps.

The clustering of differential visual responses into spatial maps was among one of the earliest discoveries of visual neuroscience, but the functional significance of this clustering is still not fully understood. In many areas that respond to visual stimuli, one finds that the spatial relationships on the retina are maintained such that neurons responding to adjacent locations in space are located close together on the retina and close together on the cortex. These topographic spatial relationships can be identified in early retinotopic areas (V1–V3) using conventional BOLD fMRI measures, and a range of other techniques including optical imaging or single-cell recordings. Moreover, they constitute a recurring feature of the spatial distribution of responses in many higher visual areas (Saygin & Sereno, 2008; Sereno et al., 1995). From Shannon’s framework, the existence of these topographic maps begs the question as to whether they provide a representational format that allows other areas of the brain to localize a given stimulus. The existence of retinotopic maps may appear very informative to us as external observers, but it should be obvious that there is no a priori reason why the rest of the brain might be able to make any use of the spatial arrangement of activity on these maps (Koenderink, 1990). Indeed, how exactly could the fact that two neurons are physically close to each other in V1 be communicated to neurons in other areas of the brain? As external observers, we can see that these neurons are close together, but how could any other area of the brain be influenced by this fact? Many would argue that the presence of maps reflects optimization constraints that makes communication more efficient, but that it does not communicate anything in itself. Thus, retinotopic maps may look useful to us, but the core question is whether the rest of the brain can use the physical position of neurons in the brain as a means of informing where to guide responses or make spatial judgments. The validity of this point is further reinforced by looking at different patient groups, who seem to have intact retinotopic maps, but who are impaired in different forms of spatial judgments. In the famous brain-damaged patient DF, for example, one can reconstruct retinotopic maps in V1 (Bridge et al., 2013) which could inform an external observer as to where objects are in relation to each other, but DF is severely impaired in making judgments about allocentric spatial relationships (Schenk, 2006). Perhaps more dramatic is the recently reported case of AG, who has a massive visual field deficit (as measured behaviorally) but shows qualitatively normal retinotopic maps (Moutsiana et al., 2014). Finally, in patients with amblyopia, one can reconstruct intact retintopic maps with input to their ‘weak’ eye (Li, Dumoulin, Mansouri, & Hess, 2007), despite very poor spatial perception when presented with stimuli to that eye (Mansouri, Hansen, & Hess, 2009).

Hence, the ability to decode spatial position as an external observer does not mean that we have identified the code by which the brain represents spatial information. By now, we hope the same point is also clear when it comes to our ability to decode object properties as external observers. The fact that certain neural responses to different object types cluster in a way that might enable successful decoding does not mean that we have identified the representational format by which the brain communicates information about objecthood. However,this is not to deny that the ability to look at large scales of activity with fMRI could provide important insights into the nature of object representations and the computations used to process them. For example, Kriegeskorte and colleagues (Kriegeskorte, 2009; Kriegeskorte, Mur, & Bandettini, 2008; Nili et al., 2014) have used the ‘representational similarity’ in activity across voxels to make inferences about the representational similarity of patterns of activity evoked by different objects. In Kriegeskorte’s words, this form of analysis can provide insights into the ‘geometry’ of neural representations (Kriegeskorte & Kievit, 2013), such that, if two object have more similar patterns of activity in fMRI, then these objects could be represented in more similar ways than two objects that evoke very different patterns. This approach, therefore, potentially offers a powerful window into the nature of the computations underlying object recognition. At the same time, however, we need to remain very cautious about the conclusions we can draw from this form of analysis given that they are very dependent upon the channel over which the information is conveyed (which we do not know), subject to the manner in which responses are anatomically clustered (often not known), and are dependent on a close correspondence between the precise flow of blood to different anatomical locations (Gardner, 2010) and the relationship between BOLD and underlying neural activity (Logothetis, 2008). The potential power of this technique, however, reinforces the need to focus on testing whether ‘potential information’ measured with fMRI really is ‘actual information’ used by the brain.

## How do we test whether ‘potential information’ in fMRI (experimenter-as-receiver) is ‘actual information’ (cortex-as-receiver)?

In the previous section, we have highlighted that successful decoding reveals potential information, but that we need to go further to test whether different patterns of activity constitute actual information used by the brain, especially if those patterns are to be used to make inferences about the nature of underlying representations (Kriegeskorte, 2009; Kriegeskorte, Mur, & Bandettini, 2008; Kriegeskorte & Kievit, 2013; Nili et al., 2014) and about the computations by which representations are combined (Baeck, Wagemans, & Op de Beeck, 2013). This section will consider a range of approaches for testing whether information decoded by the ‘experimenter-as-receiver’ is actual information used by the ‘cortex-as-receiver’.

One approach to providing evidence that potential sources of information (identified with experimenter-as-receiver) are in fact information with cortex-as-receiver is to look for a trial-by-trial correlation between the performance based on fMRI-MVPA and the behavioral performance of the participant (as used in single-cell studies described in the previous section). Using this approach, Williams et al. (2007) argued that ‘only some spatial patterns of fMRI response are read out in task performance.’ Their paper started from the observation that the patterns of neural activity in a number of different areas of the brain could be used to successfully decode which of three shape categories was presented to a participant. Only one of these three areas (the Lateral Occipital Cortex, LOC) revealed a correlation between the classifications based on neural activity and the behavioral classification judgments made by the participants. In simpler terms, when the participant made a mistake, the output of the decoding based on patterns of activity in LOC often produced the same mistake. The decoding based on patterns from the ‘retinotopic cortex’^1^ also produced errors, but these did not correlate with the behavior of the participant. Based on this finding, the authors argued that, whilst many areas may contain informative patterns of activation, only the pattern of activation present in a particular area (LOC in this case) is actually ‘read out’ or used in forming the participant’s behavioral response. By explicitly testing the link between decoding performance and behavioral performance, this study constitutes an important advance on many MVPA studies in providing more evidence that the activity in a given area of the brain really is information with cortex-as-receiver. At the same time, however, it raises some challenging questions if their logic is followed. Are we to assume that the behavior of the participant is literally based on a linear classification of the patterns of activity in LOC? If this is the case, what area of the brain is able to act as a receiver integrating signals from the whole of LOC (used to identify the potential information with fMRI)? The lack of a correlation between the patterns in ‘retinotopic cortex’ with behavior also leaves open many questions: Are these patterns in retinotopic areas a mere consequence of the way representations cluster (like those seen in spatial maps) that plays no role in the computation of shape? Or are the large-scale patterns evident in early areas directly used (transformed in some way) to construct representations of shape in intermediate visual areas?

Thus, whilst a correlation with behavior is a useful first step (and could be used more routinely in fMRI-MVPA research), we still need to focus on ‘what causes what’ in the brain to be sure that decoding with fMRI reveals information used by the brain. V1 is a perfect case in point because, although many higher areas feed-back to V1, V1 does not directly project to areas that are likely to be involved in making a final decision (Kravitz et al., 2013). Thus, for V1, the pertinent question is often not whether it directly drives (and thus correlates with) behavior, but whether it drives activity in other intermediate areas of the brain such as V2 or MT.

Research has already attempted to use dynamic causal modeling to analyze the effective connectivity between V1 and MT (Haynes, Driver & Rees, 2005) and V1 and V2 and MT/V5 (Friston & Buchel, 2000). Indeed, studying the ‘big data’ of these ‘functional interactions’ is clearly going to be a large part of the next wave of neuroscience (Turk-Browne, 2013). It is important to be clear, however, that, just as neuroimaging does not provide a direct measure of information, so too dynamic causal modeling techniques do not provide a direct measure of the information transfer between two areas of the brain. These two points are intimately linked. For example, it might be that, in order to drive a response in MT, neurons in V1 have to fire at a particular phase. In this case, the rate of activity in V1 (presumably driving most of the changes in the BOLD response measured in fMRI) may not correlate with the BOLD signal in MT. Looking at effective connectivity (if signals are carried by the precise phase of neural firing) with fMRI might, therefore, lead one to miss causal connections that are in fact present.

Any attempt to use ‘big data’ in mapping ‘functional interactions’ therefore has to start from the understanding that we cannot objectively measure information, and that we do not yet know the channel over which the brain communicates information. With this perspective in mind, we think that attempts to model connectivity between different areas should focus not just on modeling the relationship between activity in different areas but critically to test *which aspect of neural activity* in one area predicts *which aspects of activity* in another. For example, in looking at the relationship between V1 and MT, one should not just model the relationship between the levels of activity in voxels in each area but also test whether the performance of a linear classifier of the activity in V1 can provide a better predictor of subsequent activity (or patterns of activity) in MT. In this way, one could more directly test whether information in V1 (with MT as the receiver) exists in the overall rate of activity or in a more complicated distributed pattern across V1. Of course, fMRI can only measure certain aspects of neural activity, and ideally fMRI needs to be combined with other techniques which can sample a broader range of candidate channels over which activity could be conveyed. For example, larger-scale multi-electrode recordings could not only enable one to develop models based on average firing rates (and certain large-scale population codes) but also on more subtle timing and population codes. The technical feasibility of such recordings is also rapidly improving. For example, Port, Sommer and Wurtz (2000) and Port and Wurtz (2003) have reported data from simultaneously recorded neurons in different parts of the Superior Colliculus, and more recent research has also shown that measuring from larger collections of neurons can provide data that can enable one to predict the activity of individual neurons (Stevenson et al., 2012). Moreover, the number of neurons that can be recorded in one area is also rapidly increasing (Stevenson & Kording, 2011). Of course, large-scale multi-electrode recordings might also miss representations on scales that would be more easily detected with fMRI, so we do not wish to promote one technique over the other per se, but rather highlight the importance of being able to simultaneously test different potential candidate channels over which information might be conveyed by looking at which best predicts subsequent activity in a receiver.

There is one recent study which illustrates very clearly the value of looking not just at the transfer of activity from one area to another but which explicitly tests what aspect of neural activity in one area predicts activity in another. Van Kerkoerle et al. (2014) recorded simultaneously from V1 and V4 and showed that feedforward communication from V1 to V4 was based on activity in a higher frequency (gamma) range, whilst feedback (from V4 to V1) was based on lower frequency (alpha) activity. This study offers a major advance in highlighting that different temporal codes may be used as the channels over which different types of information are conveyed. Perhaps most impressively, van Kerkoerle et al. were able to show that inducing higher frequency activity in V1 caused more activity in V4, whilst in V4 inducing lower frequency activity caused more activity in V1. Thus, van Kerkoerle et al. tested not only whether activity in one area was related to activity in another but also what aspect of that activity was critical, with a specific receiver in mind.

The study by van Kerkoerle et al. (2014) also goes beyond most studies using TMS and neuropsychology in testing not just whether an area of the brain is critical but also in explicitly testing what aspect of activity in a given area is critical with a specific receiver in mind. Typically, the use of TMS or data from neuropsychology patients can add to our confidence that responses measured in a given area are (or are not) causally important, but they do not necessarily help to clarify which aspect of neural activity is actually critical in conveying information. A typical brain lesion or standard TMS intervention will undoubtedly disrupt all of the possible coding channels one could consider (firing rates at different frequencies, the phase of neural activity, population codes, etc.). However, using TMS, there are also examples where time-locked interventions could prove much more powerful in establishing not only that an area is causally involved but also in helping to identify what neural code is being used to communicate information. For example, Romei, Driver, Schyns and Thut (2011) showed that TMS applied to the same area of the brain at different frequencies had different effects on perceptual processing. More specifically, they found that higher-frequency stimulation led to more local perceptual performance on a Navon (1977) task, and lower-frequency stimulation led to more global perceptual performance. This result not only adds to the evidence that this area of the brain is causally involved in this perceptual process, but also provides evidence regarding the code by which this area computes information. Used in this way, TMS (and other tools for manipulating the temporal properties of neural firing) can advance on neuroimaging methods, by not only providing evidence that an area is active during in a particular task but also by helping to test how neural activity in a given area is actually used to communicate information.

Before concluding this section on fMRI, it is worth noting that, whilst most neuroscience is really interested in information with cortex-as-receiver, there are contexts in which it is quite appropriate to use fMRI with the explicit purpose of using neural activations to inform the experimenter (experimenter-as-receiver). Examples include trying to identify if somebody is lying (Ganis, Kosslyn, Stose, Thompson, & Yurgelun-Todd, 2003), trying to use neural activity to assess whether a coma patient is still conscious (Owen et al., 2006), or reconstructing what a participant is watching (Nishimoto et al., 2011). The practical utility of these approaches is to some extent (although not totally) separate from the issue of whether one is recording activity that actually reflects information (differences that make a difference) to the brain itself. In these instances, the physical measurements can immediately be regarded as information in Shannon’s framework because the act of communication can be defined such that the participant’s brain is the transmitter, the BOLD measured via fMRI is the channel, and the experimenter is the receiver.

## Is information carried by ERPs, rhythms, phase or traveling waves?

The use of terminology that suggests or implies that neuroimaging can directly measure the neural code or neural representations is not limited to fMRI or single-cell recordings. An interesting case in point here is a paper with the title ‘Cracking the code of oscillatory activity’ (Schyns et al., 2011). This paper looks at oscillatory rhythms during a task requiring the perception of facial emotion and suggests that more information is carried in the phase of those rhythms than in their frequency or amplitude. Within Shannon’s framework, in which information needs a transmitter, a channel and a receiver, this work implicitly gives the role of the receiver to the experimenter. However, as with other examples cited, this does not mean this work is not important or useful; on the contrary, it focuses on a very important question regarding which physical differences carry potential information. Whilst most research assumes that information is carried in the power of the signals recorded in MEG, this study highlights that more information might be carried in the phase of neural activity. However, whether thinking about phase or power, the key question should be whether any area of the brain can receive or ‘observe’ the physical signal that we measure with MEG. Stepping back, we could ask more generally whether the physical differences measured with EEG or MEG reflect or derive from physical differences that make a difference for further processes in the brain.

MEG and EEG measure signals that arise with the coordinated activity of millions of neurons. It has long been supposed that this coordinated activity is intimately tied to cognition, but it has also been argued to be epiphenomenal (Collura, 2013; Freeman, 1995). Just as steam might rise from a factory, but not contribute to the actual process of production, neurons firing might also generate electromagnetic fields that have no influence in their own right. Recent evidence from in vitro experiments, however, suggests that the responses of cortical and hippocampal neurons actually can be influenced by the surrounding, endogenous electromagnetic field (Fröhlich & McCormick, 2010). In principle, therefore, these large-scale electromagnetic fields might play a direct role in the transmission of information.

The question still remains, however, as to whether it is helpful to already refer to this physical difference as ‘information’ in the brain, when we do not yet have a model regarding how the brain could possibly use this as a signal in further processing. This is not simply an issue of making more conservative conclusions, it has important consequences in guiding future research. A good illustration of this point involves the way event-related potentials (ERPs) are computed and used. Decades of study have suggested that the averaged, time-locked EEG responses to stimuli provide a direct insight into the underlying representations. ERPs are assumed to provide a direct measure of mental chronometry (Posner, 2005) and information processing (Näätänen, 1990). Recent work looking at traveling waves of activity across the cortex suggests, however, that late ERPs (e.g., P2–N2, P3) do not emerge from a static cortical dipole, but might instead be a by-product of the way different traveling waves are combined across trials (Alexander, Trengove, Wright, Boord, & Gordon, 2006; Alexander et al., 2013). More specifically, this work suggests that most of the phase variability at the single trial level can be explained as traveling waves. This leads to the potential interpretation that in MEG, EEG and ECoG, static, localized regions of activation, with peaks at specific latencies, are actually a by-product of experimenter-averaging of waves travelling in a variety of directions. ERPs (and event-related fields and trial-averaged local field potentials) are therefore potentially a prime example of how we can interpret activity with an experimenter-as-receiver perspective, when this might not be accessible to the participant’s brain, which has no direct access to the trial-averages computed by the experimenter. It is not the aim of this article to resolve how information is represented in the cortex—as dynamic traveling waves or static patterns of activity in particular areas, or as some combination—but rather to highlight that establishing which of these is true should be the core focus of neuroscience. Calling physical activations ‘information’ before we have established how (or whether) the brain can make use of that physical activation potentially distracts us from that goal.

The distinction between static/averaged ERPs and travelling waves also provides an important illustration of the risks of prematurely labeling some physical responses as noise and others as signal. In particular, averaging over trials to calculate ERPs is often justified as a means of averaging over noise, to enhance the recorded signal. However, if traveling waves actually reflect the functional topology of dynamic cortical activity, then averaging the raw brain signals over trials is inappropriate. Averaging could cause one to cancel out what might in fact be important signals. Thus, the relationship between traveling waves and ERPs illustrates that signal and noise are also very much model-dependent. It is commonly assumed that signals that are not captured by the event-locked response can be treated as random background noise (Arieli, Sterkin, Grinvald, & Aertsen, 1996; Gruber, Klimesch, Sauseng, & Doppelmayr, 2005). However, for the case of waves travelling in different directions, the lack of cross-trial synchrony does not mean that there were no coherent patterns of phase within each trial, which might be lost when looking at the trial average.

The potential pitfalls of focusing on trial-averaged evoked responses, and the bias this can lead to in interpreting what is signal and noise, are illustrated in a study by Arieli, Sterkin, Grinvald, and Aertsen (1996), who investigated the topography of local field potentials in cat visual cortex. They found that the spatial pattern of within-trial activity dominated the signals they recorded, whilst the trial-averaged evoked responses contributed very little. Critically, they measured the activity before (and during) the usual latency window of the evoked response and found that the preceding within-trial activity was an excellent predictor of within-trial activity at the latency of the evoked response―in fact, a much better predictor than the trial-averaged evoked-response topography. This means that the within-trial topography of activity is in fact highly structured, whilst it would normally be regarded as (to-be-averaged-away) noise in the background of the assumed signal of the trial-averaged evoked response (Arieli et al., 1996).

## Is the world a transmitter? What is the channel? Who is the real homunculus?

Shannon’s theory of information specifies a transmitter, a channel and a receiver. This formulation is useful in that it forces one to be more explicit about the assumptions one is making about how neural activity can be interpreted. However, it is also questionable as a model for thinking about what perception and cognition actually do. In particular, if we consider sensory processing, it might seem strange to consider the physical world as a ‘transmitter’, the visual system as a ‘channel’ and the motor cortex (or areas involved in making perceptual decisions) as a ‘receiver’, particularly because it is arguable that certain behavioral responses are driven by representations that have no inherent basis ‘in’ the physics of the external world (Hoffman, 2009; Koenderink, 2014; Rogers, 2014). Our cortex might represent certain signals as belonging to the same object, for example, but it is philosophically questionable whether that object can be said to exist in any objectively definable way in the actual physics of the world, as a pre-existing signal that was ‘sent’ by the transmitter. In distinguishing (segmenting) discrete objects, one could argue that perception is not so much an act of ‘communicating’ (object) information but an act of ‘creating’ (object) information. That is to say, perception could be understood as the process of creating differences that make a difference, that exist only because of what the brain does. Thus, if perception should be understood as the construction of information as part of a ‘user-interface’ that enables us to interact with the world (Hoffman, Singh, & Prakash, 2015), this could suggest a profoundly different framework for thinking about information than that established by Shannon. This potential distinction between communicating information and creating information is just one reason why Shannon’s formulation might be a questionable framework for thinking about information processing in the cortex. Again, however, Shannon’s precise formulation brings into clear focus a fundamental question for neuroscience that might otherwise be neglected.

Another complication to Shannon’s formulation is the hierarchical but interactive nature of cortical dynamics, which means that a clear segregation into a transmitter, channel and receiver maybe inappropriate. Particularly if the cortex implements some form of predictive coding, information then becomes a complex interaction between the receiver and transmitter, which could result in only ‘error signals’ being conveyed over the channel. Shannon’s initial formulation does not consider more complicated feedback or recurrent dynamics. These considerations are not straightforward or easy, but, again, these are not side issues that neuroscience can ignore while it gets on with the business of measuring information in the brain. Instead, these are fundamental considerations that need to be considered from the outset.

There are unarguably many candidate channels over which information could be conveyed in the cortex, but a lot of neuroscience is conducted with the assumption that we already know what that channel is. The first sentence of a recent *Nature Neuroscience* publication (Goris, Movshon, & Simoncelli, 2014) illustrates this point quite clearly: ‘Neurons transmit information with sequences of action potentials’. In another recent example, an *Annual Review of Neuroscience* article opens with the sentence ‘Information is encoded in patterns of neural activity’ (Haxby, Connolly, & Guntupalli, 2014). Both these statements are questionable, and neither is accompanied by a reference. As we have already discussed, some authors have explicitly argued that in some contexts information is not conveyed by *sequences* of action potentials but by the precise timing of the first action potential (Thorpe et al., 2001). Others have highlighted that it is plausible that in some contexts single neurons could directly code for information, and that a distributed *pattern of neural activity* might not be needed (Bowers, 2009).

Related to this, neuroscientists have long been confident in labeling some neural activity as noise, or mere ‘background’ activity. However, without an accepted definition of a channel, how can we possibly know a priori what is noise and what is signal? Indeed, the recent *Nature Neuroscience* paper cited above helps to clarify that signals previously considered to be noise might actually relate to ongoing activity, which appears as noise simply because it is not stimulus-dependent (Goris et al., 2014). Indeed, others have already highlighted the potential dangers of selectively recording from neurons that are pre-identified as behaving ‘rationally’ or excluding those (in the study of perception) that seem at first glance ‘visually unresponsive’ (Olshausen & Field, 2005). The problem with this pre-selection is again that certain neurons may appear (un)informative to us, but the functional role of these neurons is not to communicate information to an external observer but to communicate with the rest of the brain. It could very well be that the neurons with the non-obvious or less reliable responses are actually providing a critical signal to the rest of the brain. There is a very real risk that we will never be able to build or test valid computational models (which might actually inform us about what really is signal and noise for the cortex), if the signals recorded are biased towards those that made more intuitive sense to the experimenter.

In an earlier section, we questioned the terminology used in a paper purporting to demonstrate that phase carries more information than power in MEG. It is useful to clarify here that our concern with this research is that it draws a conclusion about information with cortex-as-receiver based on what can be deciphered with the experimenter-as-receiver. However, despite this shortcoming, this work is actually more directly focused on what we would regard as the most immediate and important challenge of neuroscience, namely, focusing on how information might be conveyed (via power or phase) rather than just assuming this is something we already know. The work of Singer and colleagues is another prime example here, where there is a clear attempt to address whether more information could potentially be conveyed in spike timing rather than in rates of firing (Singer, 1999).

One might argue that focusing on how one area of the brain can act as a ‘receiver’ for signals from another area of the brain returns us to the idea of a naive homunculus which ‘observes’ neural activity and makes decisions about it. Often, however, the dreaded homunculus has in fact been replaced, not with a theory of information processing but by an external experimenter who observes and interprets recorded activity. This assumption is sometimes embedded in sophisticated mathematical frameworks such as the computation of mutual information between activity recorded by an external observer and stimuli or conditions defined by that observer. As in the example of looking at phase and power in MEG, the use of these techniques certainly provides a powerful tool for actually quantifying what are potentially informative signals. Once identified, however, we need to move from the fact that these signals can be identified by an external observer to thinking about how they could actually be used as a neural code. There may not be a homunculus in the brain interpreting neural signals, but, if one presents a visual discrimination task to a participant, somehow the responses in visual areas need to be communicated to the motor cortex making a response. It is certainly useful to directly record the responses in V1 in such an experiment, but one cannot assume that the firing of a neuron, or the decoding of a pattern which can inform one as an external observer, is the same physical response that will be used by other areas of the cortex to eventually initiate the correct motor response. Rather than simply correlating between one physical activity and presented stimuli, one needs to record a rich range of physical responses in V1 that could act as potential signals, and then track the causal dynamics from one area to the next to see which of these best predicts whether physical activity is evoked in the next area of the cortex. This way of doing research is not an entirely novel endeavor. As discussed above, there are already attempts to predict activity in one area, from activity in another . For example, in a recent study, Heinzle, Kahnt, and Haynes (2011) looked at the topographic relationships between activations in V1 and V3, and found a clear mapping between the location activated in V1 and the corresponding topographic locations in V3. Interestingly, they also found that the topographic specificity, in terms of whether a response in V1 would be correlated with a response in V3, was also observed when the participant was in a completely dark room. Thus, spontaneous activity seemed to be structured in a way that suggests that it is not simply ‘background noise’ but that it might play a functional role.

Tracking causal dynamics between areas of the brain in order to make inferences about what physical responses might act as sources of information is clearly not an easy task. We would argue, however, that a lot of modern neuroscience is difficult to interpret if we assume (often implicitly) that we have already established what is signal and what is noise. If we do not yet know what physical responses can be used as information, it will clearly be hard, if not impossible, to answer some of the questions that are the focus of modern neuroscience. How, for example, can we really know whether a given area only represents faces when we do not actually know how the brain represents information? If this paper is to prove informative to the field, we hope that it makes a difference not only in the extent to which we are explicit in the assumptions we make when reporting neuroimaging data but that it might also encourage a greater focus in tracking causal dynamics in the brain in a way that enables us to identify the differences that make a difference in the brain.

## Of straw men and neuroimaging: what are the implications for neuroscience?

To many researchers, the idea that neuroimaging might be assumed to provide a direct measure of information is so obviously false that questioning it might seem like nothing but a straw man argument. At the same time, however, results in neuroimaging (and especially the recent rise in studies using fMRI-MVPA) are often communicated as discoveries of the ‘neural representation of X’, or the ‘neural encoding of Y’. As we noted earlier, some of the earliest work linking neural responses to behavior was much more straightforward in communicating findings from neuroimaging, referring (for example) to the ‘neural correlates of a perceptual decision’ (Newsome et al., 1989). The terminology of ‘neural representations’ or ‘codes’ might not be problematic if it was clear to everyone that these terms are used as a short-hand, or proxy, for a neural response which could potentially provide information to other areas of the brain. However, if the terminology of ‘neural codes’ and ‘neural representations’ is used, one should be able to cite existing literature that clarifies its meaning. Particularly as neuroimaging is adopted by diverse fields from neuro-law to neuro-economics, the imperative to make explicit assumptions that might be clear only within a certain academic community becomes even more important. Although we do not wish to claim that Shannon’s framework is the definitive and only viable way to think about information in the brain, we think its emphasis on a receiver helps to bring into clearer focus what neuroimaging can measure, and how that can be communicated more clearly. Within neuroscience, we hope this article will cause a shift in emphasis away from thinking about what we can decode from different neuroimaging techniques (with experimenter-as-receiver) to thinking about whether those recordings of neural activity are differences (or markers of underling differences) that could be decoded by the rest of the brain. Although the technical challenges would still be substantial, we hope this article makes the theoretical case that it is imperative to simultaneously record from different areas of the brain to test which aspects of neural activity are actually communicated from one area to another to establish what actually constitutes information in the brain. Recording from areas like V1, and in areas sensitive to the same part of the visual field in areas receiving inputs from V1 (such as V2 or MT), with multi-electrode arrays would be an ideal starting point. By recording simultaneously whilst an organism is performing a visual task, it would become possible to test which aspect of V1's activity best predicts subsequent activity in areas receiving that input. By looking at a transmitter (such as V1) and a receiver (such as V2 or MT), we can make progress in identifying the channel over which information is actually conveyed. Such an analysis could have profound implications. If, for example, it was found that most of the activity in V2 or MT was driven by the precise timing of firing in V1, rather than by the overall rate at which neurons fire, then we would have to seriously re-evaluate the contribution that studies using fMRI could make to our understanding of information in V1. Particularly given the recent increase in studies using fMRI-MVPA (which sometimes explicitly claim to decode information in the brain), we hope that this paper offers a timely critique of assumptions that could lead psychologists to use neuroimaging to ask questions that neuroimaging cannot yet answer. Moreover, we hope that this paper will act as a catalyst to neuroscience to focus on the development of methods and techniques that enable us to study what causes what in the brain, and thus start to unravel the neural code.

## Footnotes

1 ‘Retinotopic cortex’ is in quotes here because evidence has accumulated since that LOC is also organized into a number of retinotopic maps of spatial relationships (Larsson & Heeger, 2006), and is therefore also ‘retinotopic’.
